# Job performance among health professionals in Ethiopia: a systematic review and meta-analysis

**DOI:** 10.3389/phrs.2026.1609470

**Published:** 2026-06-17

**Authors:** Hailemariam Gezie, Mekuriaw Wuhib, Dagnew Tigabu, Habtam Gelaye

**Affiliations:** 1 Department of Emergency and Critical Care Nursing, College of Health Sciences, Debre Tabor University, Debre Tabor, Ethiopia; 2 Department of Comprehensive Nursing, College of Medicine and Health Sciences, Wollo University, Dessie, Ethiopia; 3 Department of Pediatrics and Child Health Nursing, College of Medicine and Health Sciences, Woldia University, Woldia, Ethiopia; 4 Department of Psychiatry, College of Medicine and Health Sciences, Wollo University, Dessie, Ethiopia

**Keywords:** Ethiopia, health professionals, job-performance, nurses, work-performance

## Abstract

**Objectives:**

This systematic review and meta-analysis aimed to explore the pooled estimate of good job performance among health professionals in Ethiopia.

**Methods:**

We searched multiple databases, such as PubMed, African Journals Online, Hinari, Google Scholar, and repositories, by combining keywords using the Boolean operators “AND” and “OR.” Observational studies that reported job performance were screened by their title, abstract, and full texts and included in this review. Data were extracted using a standardized Excel template and analyzed using STATA 17 software. We pooled good job performance in the random effects model. Heterogeneity was checked using the Galbraith plot, I^2^, and Q statistics and identified by subgroup and sensitivity analyses. Publication bias was assessed using a funnel plot.

**Results:**

The meta-analysis revealed a 66% (CI: 55, 77) pooled prevalence of good job performance among Ethiopian health professionals. Higher estimates were observed in studies with a sample size of <350 (72%), conducted in Addis Ababa (76%), after 2020 (65%), and among nurses (69%).

**Conclusion:**

Only two-thirds of Ethiopian health professionals demonstrated good job performance, highlighting the need for implementing targeted performance improvement interventions.

**Systematic Review Registration:**

https://www.crd.york.ac.uk/PROSPERO/view/CRD420251013239, identifier CRD420251013239.

## Introduction

The job performance of health professionals is one of the critical factors that influence a country’s healthcare system [1]. Job performance is a multidimensional construct encompassing different aspects of employee behavior that contribute to organizational effectiveness, including task performance, contextual performance, adaptive performance, and counterproductive work behavior [1, 2] Competent and skilled health professionals, through both individual and collective contributions to organizational goals, are essential for delivering efficient, high-quality, and equitable health services and achieving desired health outcomes [3–5]. Poor performance among healthcare providers can lead to inaccessible and inappropriate care, mistreatment of clients, reduced healthcare quality, and poor health outcomes [6].

A range of factors can influence the job performance of health professionals. These include individual factors (e.g., age, gender, educational level, personality, work experience, motivation, job satisfaction, burnout, and self-efficacy), organizational factors (e.g., organizational culture, leadership style, work environment, workload, resource availability, supervision, mentorship, and professional development opportunities), and system-level factors (e.g., health policies, regulations, and financing issues) [3, 7–16].

Previous literature indicates that job performance among health professionals varies across countries. For instance, studies conducted in Pakistan and Indonesia reported that 60.6% of 360 medical doctors [17] and 40% of 50 nurses [18], 41.5% of 200 nurses [19], and 72.5% of 40 nurses [20] demonstrated good job performance. Similarly, a study conducted in Saudi Arabia found that 53.7% of 637 nurses had good job performance [21], while studies conducted in Nigeria reported that 66.78% of 849 healthcare workers [22] and 80% of 490 healthcare workers [23] had good job performance.

The Ethiopian healthcare system is facing multiple challenges in providing quality healthcare due to its fast-increasing population and subsequent increasing health service requirements [24]. The availability of qualified and high-performing health professionals is among the problems. To reduce job performance-related problems, the Ethiopian health system, led by the federal ministry of health, has system-wide performance appraisal methods such as the balanced scorecard, job evaluation and grading, supportive supervision and mentorships, continuous capacity building and skill development opportunities, and motivational incentives or rewards to create a performance-oriented culture in the health sector [25–27]. There have also been recent advancements, including digital platforms and innovations that support the implementation of digital health. These job performance management techniques are executed through different strategies, such as standard-based management; decentralizing governance to regions, zones, and woredas to enhance ownership and responsiveness among local health leaders; using performance measurements and key performance indicators; using continuous and periodic performance review systems; and data-driven decision-making [28, 29]. Additionally, the Ethiopian health sector has made significant efforts, such as integrating these strategies into national policies and annual plans, strengthening leadership capacity, and promoting digital innovation to institutionalize their implementation [29].

Despite the adoption of a variety of job performance management strategies and implementation efforts to increase healthcare access, coverage, infrastructure, and human resource development over the past few decades, job performance among Ethiopian health professionals remains suboptimal. Addressing these challenges requires that governmental and stakeholder efforts be supported by evidence generated through rigorous research conducted by scholars and experts. Although some primary studies have explored the job performance of health professionals in Ethiopia, there is a lack of pooled evidence summarizing the existing findings. Addressing this gap could be beneficial for health leaders, policymakers, health planners, and health facility leaders. Therefore, this systematic review and meta-analysis aimed to fill the gap by estimating the pooled prevalence of good job performance among health professionals in Ethiopia.

### Review questions

- What is the prevalence of good job performance in health professionals in Ethiopia?
- What are the predictors of good job performance in health professionals in Ethiopia?

## Methods

### Study design and registration protocol

This systematic review and meta-analysis was designed based on the Preferred Reporting Items for Systematic Reviews and Meta-Analysis (PRISMA-2020) checklist [30]. The review protocol was registered in PROSPERO with the registration number CRD420251013239, which is available at https://www.crd.york.ac.uk/PROSPERO/view/CRD420251013239. No amendment was made to the protocol after registration in Prospero.

### Data source and search strategy

To ensure a wider coverage of relevant studies, we searched multiple databases and search engines: PubMed, African Journals Online, Hinari, Google Scholar, and repositories. A snowball search for references of included studies was also performed. MeSH (Medical Subject Heading) words and phrases were combined to create a search strategy for each database. The following search terms were used for PubMed: (((((((((“Job performance”[Title/Abstract]) OR (“Work performance”[Title/Abstract])) OR (“Performance at work”[Title/Abstract])) OR (“Health professionals performance”[Title/Abstract])) AND (“Associated factors”[Title/Abstract])) OR (predictors[Title/Abstract])) OR (determinants[Title/Abstract])) AND (“Health professionals”[Title/Abstract])) OR (“Healthcare workers”[Title/Abstract])) AND (Ethiopia [Title/Abstract]). For African Journals Online, Google Scholar, and manual searches, we used the following combination: “Job performance” OR “Work performance” OR “Performance at work” OR “Health professionals’ performance” AND “Associated factors” OR predictors OR determinants AND “Health professionals” OR “Healthcare workers” AND Ethiopia. For Hinari, we used the search terms “Job performance among health professionals in Ethiopia” (Table 1).

**TABLE 1:** Search strategy of databases, registers, and repositories, Ethiopia, 2025 (n = 663 articles).

Data bases, register	Search string	Search date	Search result	Inclusion & exclusion criteria	Framework
PubMed	(((((((((“Job performance”[Title/Abstract]) OR (“work performance”[Title/Abstract])) OR (“Performance at work”[Title/Abstract])) OR (“Health professionals performance”[Title/Abstract])) AND (“Associated factors”[Title/Abstract])) OR (predictors[Title/Abstract])) OR (determinants[Title/Abstract])) AND (“Health professionals”[Title/Abstract])) OR (“Healthcare workers”[Title/Abstract])) AND (Ethiopia [Title/Abstract])	December 10, 2025	574	Inclusion criteria • Studies conducted among health professionals or healthcare workers in Ethiopia • Observational studies reporting the prevalence of good job performance and its predictors such as cross-sectional, cohort, and case-control studies • Articles published in English and unpublished studies Exclusion criteria • Studies conducted outside Ethiopia • Observational studies not reporting the prevalence of good job performance and its predictors such as qualitative studies, reviews, editorials, and commentaries • Studies with no full-text access after reasonable attempts • Duplicate Studies or systematic reviews and meta-analyses	Population: Health professionals in Ethiopia Exposure: Working within the Ethiopian healthcare system Comparison: None/not applicable Outcome: Job performance level
African Journals Online	“Job performance” OR “work performance” OR “Performance at work” OR “Health professionals’ performance” AND “Associated factors” OR predictors OR determinants AND “Health professionals” OR “Healthcare workers” AND Ethiopia	December 10, 2025	20		
Google Scholar			21		
Manual search from google			16		
HINARI	“Job performance among health professionals in Ethiopia”	December 10, 2025	28		
Repositories			6		

### Eligibility criteria

All studies published in English that reported job performance and associated factors among health professionals and were published until December 10, 2025 and gray literature, such as master’s theses and doctoral dissertations available online until this date, were included in this review. This date limit was determined by the authors based on the schedule of the protocol development and registration process.

Observational studies such as cross-sectional, case-control, and cohort studies conducted in Ethiopia were included. Studies focused on particular demographic characteristics, case reports, case series, letters to the editor, studies that did not report the prevalence of blood donation practices, and meta-analyses were excluded.

### Outcome of the review

The outcome variable of this review is the job performance among health professionals in Ethiopia. Job performance was categorized as “good” or “poor.” Participants who scored greater than or equal to the mean on self‐rated Likert scale assessment tools were categorized as having “good performance,” while those who scored below the mean were categorized as having “poor performance” in the primary students.

### Study selection and screening

All the studies searched for using various databases and search engines were exported into Endnote X9, and duplicate articles were removed. Then the title, abstract, and full text of each article were examined independently by three authors (HmG, DT, and MW). Disagreements between the three authors were resolved through discussion with the fourth author (HG).

### Data extraction and data items

Data were extracted from each included study using a standardized data extraction template prepared in Microsoft Excel. Two authors (HG and MW) extracted information such as the last name of each author, year of publication, location of studies, type of profession, response rate, sampling technique, sample size, the proportion of good job performance (P), and associated factors in odds ratio (OR) with their 95% confidence interval independently. Discrepancies between the two authors were resolved by discussing with the third author (HmG).

### Risk of bias assessment

The Joanna Briggs Institute (JBI) quality appraisal tool, which was modified for observational studies, was used to assess the quality of the included studies [31]. The tool has eight items. The first item was about criteria for inclusion in the sample; the second was about study subjects and the setting; the third was about exposure measurement; the fourth was about measurement of the condition; the fifth was about confounding factors; the sixth was about strategies to deal with confounding factors; the seventh was about outcomes measurement; and the eighth was about statistical analysis used with responses “Yes,” “No,” “Unclear,” or “Not Applicable.” It was assessed by two reviewers (DT and MW) independently. Any discrepancy between the three reviewers’ results was resolved by discussion with the third reviewer (HmG).

### Data analysis, certainty assessment, and publication bias assessment

After extracting the data using Microsoft Excel format, it was exported to STATA version 17 for analysis. The pooled estimate of job performance among health professionals in Ethiopia was estimated using DerSemonian and Liard’s method in the random effects model [32]. The pooled estimate of good job performance was presented using a forest plot. Statistical heterogeneity between the included studies was identified using the Galbraith plot, I^2^, and Q statistics [33]. A subgroup analysis by publication was performed by publication period, region of study, sampling technique, type of profession, response rate, sample size, and study quality to identify the source of heterogeneity. Sensitivity analysis was also done to assess the robustness of the findings. Publication bias was assessed using visualization of a funnel plot [34]. A narrative review of factors associated with job performance among health professionals in Ethiopia was also conducted.

## Results

### Study selection

**FIGURE 1:**
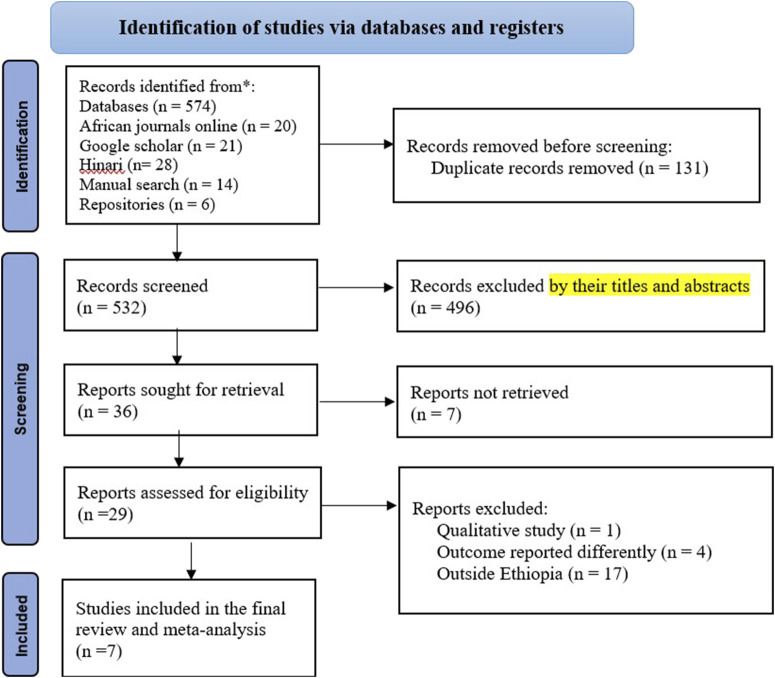
The Preferred Reporting Items for Systematic Reviews and Meta-Analysis 2020 flow diagram for a systematic review and meta-analysis of job performance among health professionals in Ethiopia, 2025.

Six hundred sixty-three (663) articles were found in the initial search of both published articles and gray literature until December 10, 2025. Among these articles, 574 were from PubMed, 20 were from African journals online, 21 were from Google Scholar, 28 were from Hinari, 16 were from manual search, and six were from repositories. These documents were imported into the EndNote X9 citation manager. Of the total articles searched, 131 were found to be duplicates and removed. The other 496 articles were found irrelevant and excluded because their titles and abstracts were unrelated to the job performance of health professionals, and 36 articles were retrieved for full text. Finally, seven studies that included 2170 health professionals fulfilled the inclusion criteria and were included in the final meta-analysis (Figure 1).

### Characteristics of included studies

Of the seven articles, two studies were conducted in the Amhara regional state [35, 36], two were conducted in the Oromia regional state [10, 37], and two were conducted in AA [38, 39], and one study was conducted in the central Ethiopia regional state [40]. Regarding the sampling method, the majority (four) of the studies used a simple random sampling technique [10, 35, 37, 38], and the other three studies used multistage [40], census [36], and stratified [39] techniques of sampling, respectively. Five studies were conducted among all health professionals [10, 35, 36, 39, 40], and the other two studies were conducted among nurses only [37, 38] (Table 2).

**TABLE 2:** Characteristics of the studies included in the systematic review and meta-analysis, Ethiopia, 2025.

Author Publication year	Location (region of studies)	Sampling technique	Profession	Sample size	Good perf-romance	Prevalence	Quality score
Daba L et al. [38]	AA	SRS	Nurse	166	117	0.705	7
Tesfaye T et al. [37]	Oromia	SRS	Nurse	239	162	0.678	5
Ousman Y et al. [10]	Oromia	SRS	All*	381	184	0.482	6
Bereda S et al. [36]	Amhara	Census	All*	103	69	0.67	7
Kamiso BD et al. [40]	Central Ethiopia	Multistage	All*	617	302	0.4895	5
Bewket AG et al. [35]	Amhara	SRS	All*	364	281	0.772	7
Tamrat T et al. [39]	AA	Stratified	All*	300	244	0.813	5

Abbreviations: All*, All Health Professionals; SRS, Simple Random Sampling; AA, Addis Ababa.

### Risk of bias among studies

The total score of included studies in the quality assessment varied from 4 to 8. Studies with a score of <4 were of low quality, studies with a score of 4–5 were of moderate quality, and studies with a score of 6–8 were of high quality. Of the seven studies, four studies were found to have low risk [10, 35, 36, 38] and the other three studies had moderate risk [37, 39, 40]. No included study was found to have high risk (Supplementary Material 1) and (Table 2).

### Pooled estimate of good job performance

**FIGURE 2:**
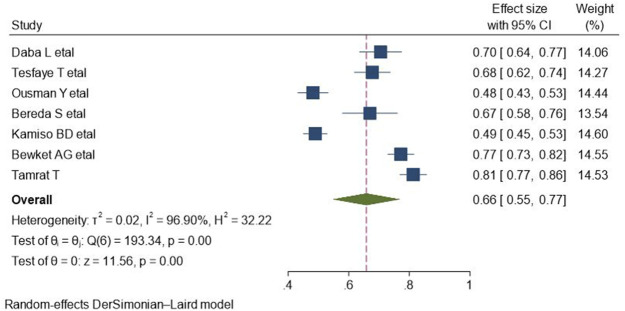
Forest plot for the pooled estimate of job performance among health professionals in Ethiopia, 2025.

This systematic review and meta-analysis revealed that the pooled estimate of job performance among health professionals was 66% (CI: 55, 77) in the six studies conducted among health professionals in Ethiopia. However, there was high heterogeneity from variations between the included studies (I^2^ = 96.90%, p = 0.00) (Figure 2).

### Identifying heterogeneity and the source of heterogeneity

Heterogeneity was checked by the Galbraith plot and I^2^ and Q statistics. To identify the source of heterogeneity, subgroup analysis and sensitivity analysis were done in the random effects model because there was high heterogeneity between studies.

### Galbraith test

The Galbraith plot was done and indicated no study was outside the confidence interval, which indicated no heterogeneity due to extreme outlier studies (Supplementary Material 2). However, the Galbraith plot within the confidence limit may not mean studies are homogeneous.

### Subgroup analysis

A subgroup analysis was also carried out for the publication period, location of studies, sampling technique, type of profession, response rate, sample size, and quality of studies. A subgroup analysis by publication year revealed that the pooled estimate of good job performance among health professionals was higher in studies conducted after 2020 (65%; CI: 51, 80) than in studies done before 2020. The subgroup analysis by location of studies indicated that the highest pooled estimate of good job performance was seen in AA, which was 76%; CI: 66, 87). Additionally, the subgroup analysis by profession revealed the highest pooled estimate among studies conducted among nurses only (69%; CI: 64, 73) compared to all professions. On the other hand, no difference was seen in the pooled estimate of good job performance from a subgroup analysis by quality of studies (Table 3).

**TABLE 3:** Sub-group analysis by publication period, region of study, sampling technique, profession, sample size, and quality of studies, Ethiopia, 2025.

Subgroup		Number of studies	Pooled prevalence (%) with 95% CI	Heterogeneity		
				I^2^	Q(DF)	P-value
Year of publication	Before 2020	2	63(63, 73)	0.00	1(0.02)	0.89
	After 2020	5	65(51 80)	97.92%	4(192.16)	0.00
Region of study	Addis Ababa	2	76(66, 87)	84.91%	1(6.63)	0.01
	Amhara	2	73(63, 83)	74.72%	1(3.96)	0.05
	Oromo	2	58(39, 77)	95.92%	1(24.49)	0.00
	Central Ethiopia	1	49(4, 53)	—	—	—
Sampling technique	Simple random	4	66(52, 80)	96.06%	3(76.22)	0.00
	Multistage	1	49(45, 53)	—	—	—
	Census	1	67(58, 76)	—	—	—
	Stratified	1	81(77, 86)	—	—	—
Profession	All professions	5	65(50, 80)	97.89%	4(189.50)	0.00
	Nurses	2	69(64, 73)	0.00%	1(0.34)	0.56
Sample size	<350	4	72(64 80)	83.27%	3(17.93)	0.00
	>350	3	58(39, 77)	98.20%	2(111.02)	0.00
Quality of studies (risk of bias)	Low risk	4	66(51, 81)	96.05%	3(75.95)	0.00
	Moderate risk	3	66(45, 87)	98.28	2(116.54)	0.00

### Sensitivity analysis

A leave-one-out sensitivity analysis was done to identify the source of heterogeneity further in the pooled estimate of good job performance among health professionals. However, there was no point-estimated prevalence of good job performance outside the confidence interval when each study was left out of the analysis. This shows that the pooled estimate of good job performance among health professionals could be plausible. The pooled prevalence in the sensitivity analysis ranged between 63% and 69% (Supplementary Material 3).

### Publication bias and evidence certainty report

**FIGURE 3:**
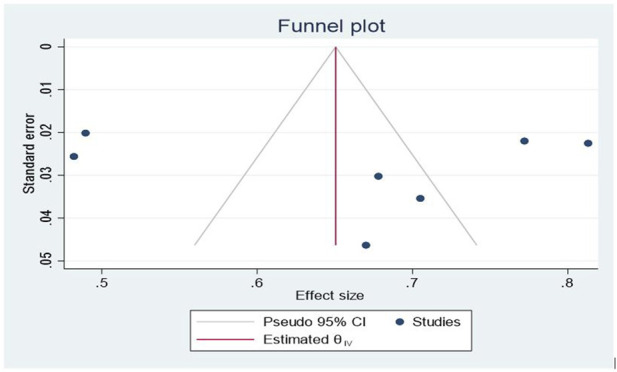
Funnel plot test to check risk of publication bias for the pooled estimate of good job performance among health professionals in Ethiopia, 2025.

To check for a publication bias, a standard funnel plot for a logit event rate of pooled estimates of good job performance among health professionals it was used (Figure 3). An Egger’s test was not applied for checking publication bias because it lacks statistical power and reliability and leads to inaccurate conclusions.

### Factors associated with good job performance

Four studies [10, 35, 37, 38] that demonstrated factors associated with good job performance among health professionals in Ethiopia were incorporated. Statements that were found to be positively associated with good job performance in a study conducted in AA were “I agree that there is sufficient staff”, “I agree that remuneration is according to experience”, “I agree that I find my work rewarding”, “I agree that the objectives to be achieved are known by individuals to be assessed”, and “I agree that feedback on how the staff is performing is provided throughout the year”. [38]. Believing that feedback on performance appraisals was good and having good self-rated knowledge and skills were found to have a positive association with good job performance in a study conducted at Jimma University Hospital [37]. Five variables (female sex, married, working for more than 8 h, good working conditions, and satisfaction with the job) were found to have an association with good job performance in a study conducted in hospitals in the West Haraghe zone [10]. Additionally, general practitioners and having good motivation were found to have a positive association with good job performance in a study conducted in public hospitals of the Awi Zone [35] (Supplementary Material 4).

## Discussion

The job performance of health professionals is one of the key determinants of healthcare quality. Therefore, this systematic review and meta-analysis summarized the job performance of health professionals and its predictors. The pooled prevalence of good job performance from the seven studies was 66% (CI: 55, 77). This indicates only two-thirds of health professionals had good job performance. However, there was high heterogeneity (I^2^ = 96.90%, p = 0.00) among included studies. This high heterogeneity might arise from methodological variations (study design, sample size, and sampling techniques), professional variations, as the studies included health professionals from different disciplines, and variations in study regions where the primary studies were conducted.

This finding was consistent with a study conducted in Nigeria, which revealed 66.78% of healthcare workers have good performance [22]. Despite many variations between Ethiopians and Nigerians, this consistency might be attributed to similarities in the profession of study participants, in which both studies included all health workers as study participants. However, the current finding was higher than findings reported by studies conducted in Pakistan (60.6%) [17], Saudi Arabia (53.7%) [21], and Indonesia (41.5%) [19], (40%) [18]. On the other hand, our finding was lower than findings from primary studies conducted in Nigeria (80%) [23] and Indonesia (72.5%) [20]. This discrepancy could be because of differences in sociodemographic and economic characteristics of health professionals, health policy and regulation variations, study design, sample size, and professional variation of study participants, in which our meta-analysis included studies conducted among any health professionals, whereas the Saudi Arabian and Indonesian studies were conducted among nurses only.

A subgroup analysis by publication year revealed the highest pooled estimate of good job performance among studies conducted since 2020 and later. This higher prevalence may be attributed to the larger number of studies and greater sample size in the included studies compared with those conducted before 2020. This may also reflect recent improvements in the healthcare system, leadership, policy, and expanded opportunities for continuous professional development for health professionals in Ethiopia. A subgroup analysis by region of studies revealed the highest pooled estimate of good job performance by studies conducted in AA compared to studies conducted in Amhara, Oromia, and the central Ethiopian regional states. The possible justification for this difference might be that AA is the capital city of Ethiopia and is referred to as the political capital of Africa, and many diplomats and businesspeople live in the city, so there are highly educated and specially trained health professionals, better health infrastructure, more health financing, and better opportunities for professional development. The work environment is also better in the city compared to remote health facilities. The Ethiopian government has also better support and regulation for health facilities found in the city. Another subgroup analysis by profession also revealed the highest pooled prevalence of good job performance by studies conducted among nurses only compared to studies conducted among all health professionals. This significant difference may be attributed to nurses’ performance usually being evaluated by specific performance metrics that are related to patient care, teamwork, and communication skills [41, 42]. Additionally, nurses spend more time with patients and have more community engagement, which may lead to improved performance. Similarly, studies with a sample size of less than 350 revealed a slightly higher pooled prevalence of good job performance compared to studies with a sample size of 350 and above. The possible justification may be that the number of studies with a sample size of <350 was greater than those with ≥350, and most of the studies with a sample size of <350 were in AA, with the higher pooled estimates compared to studies done outside AA. Despite these variations, some studies relate sample size with absolute and relative precisions, in which larger sample sizes are required to get lower prevalence rates [43]. On the other hand, the subgroup analysis by quality of studies (low vs. moderate) revealed no difference in the pooled estimate of good job performance among health professionals. This may be the shared sociocultural and economic characteristics of participants and the health system of the country.

Regarding the factors associated with job performance, a narrative review of factors revealed 14 variables were found to have a significant association with job performance in four studies. These variables could not be pooled in the meta-analysis because they were found to be significant in only one study.

Despite being the first review and meta-analysis in Ethiopia that included all published and unpublished data regarding the job performance of health professionals, this systematic review and meta-analysis had limitations, such as significant heterogeneity among studies. The review also included a very small number of studies, which could decrease the precision of the pooled estimate. Another possible limitation might be that it did not pool the predictors of job performance among health professionals and described them qualitatively because there was inconsistency in their effect size. Additionally, the cross-sectional nature of all the included studies may be another limitation.

## Conclusion and recommendations

Good job performance of health professionals is a cornerstone of the overall success of healthcare facilities in providing quality healthcare services. However, the pooled estimate from this systematic review and meta-analysis demonstrated that only two-thirds of health professionals had good job performance. Therefore, the Ethiopian Ministry of Health, regional health bureaus, zonal health departments, local government bodies, health institutions, higher education institutions, and other stakeholders should pay due attention to the improvement of the performance of health professionals. At the national level, the ministry of health should develop and implement standard performance management frameworks, strengthen continuous professional development opportunities, and allocate funding to improve healthcare infrastructure. Regional and local health systems should also establish routine supportive supervision and mentorship programs, offer performance-based financial or non-financial incentives, and reduce disparities through targeted support to low-performing areas. Additionally, healthcare facilities should implement performance evaluations, promote a conducive work environment, and optimize workload distribution to prevent burnout. Moreover, researchers should conduct qualitative, longitudinal, and intervention-based studies to establish causes of low performance and provide performance improvement strategies.

## Data Availability

The datasets that were analyzed for the current systematic meta-analysis are available from the corresponding author upon reasonable request.

## Supplementary material

SUPPLEMENTARY MATERIAL 1 Summary of risk of bias assessments using the Joanna Bridge Institute’s appraisal checklist.

SUPPLEMENTARY MATERIAL 2 Galbraith plot of the pooled estimate of good job performance among health professionals in Ethiopia.

SUPPLEMENTARY MATERIAL 3 Sensitivity analysis of the pooled estimate of good job performance among health professionals in Ethiopia.

SUPPLEMENTARY MATERIAL 4 Factor associated with good job performance among health professionals in Ethiopia.
