# Supplementary material for: Job performance among health professionals in Ethiopia: a systematic review and meta-analysis
Source: Public Health Rev. 2026 Jun 17;47:1609470. doi: 10.3389/phrs.2026.1609470 (PMC13318800; doi:10.3389/phrs.2026.1609470)
Supplement: Supplementary file 1 [file Supplementaryfile1.docx]

**Summary of quality assessments using JBI appraisal checklist**

| **Author and publication year** | **Q1** | **Q2** | **Q3** | **Q4** | **Q5** | **Q6** | **Q7** | **Q8** | **Score** |
| --- | --- | --- | --- | --- | --- | --- | --- | --- | --- |
| Daba L etal, 2024 | 1 | 1 | 1 | 0 | 1 | 1 | 1 | 1 | 7 |
| Tesfaye T etal, 2015 | 1 | 1 | 1 | 0 | 0 | 0 | 1 | 1 | 5 |
| Ousman Y etal, 2023(11) | 1 | 1 | 0 | 0 | 1 | 1 | 1 | 1 | 6 |
| Bereda S etal, 2019 | 1 | 1 | 1 | 1 | 1 | 0 | 1 | 1 | 7 |
| Kamiso BD etal, 2024 | 1 | 1 | U | 1 | NA | NA | 1 | 1 | 5 |
| Bewket AG etal, 2023 | 1 | 1 | 1 | U | 1 | U | 1 | 1 | 7 |
| Tamrat T etal 2023 | 0 | 1 | 1 | 1 | 0 | 0 | 1 | 1 | 5 |

JBI Criteria to be scored:

- Q1. Were the criteria for inclusion in the sample clearly defined?
- Q2. Were the study subjects and the setting described in detail?
- Q3. Was the exposure measured in a valid and reliable way?
- Q4. Were objective, standard criteria used for measurement of the condition?
- Q5. Were confounding factors identified?
- Q6. Were strategies to deal with confounding factors stated?
- Q7. Were the outcomes measured in a valid and reliable way?
- Q8. Was appropriate statistical analysis used?

**Abbreviations:** 1 = Yes; 0 = No; U = Unclear; NA = Not Applicable; JBI: Joanna Briggs Institute

**Criteria used to rank the risk of bias**

- <50% = high risk of Bias
- 51% - 70% = Moderate risk of Bias (2 studies)
- Above 70% = low risk of Bias (4 studies)
