# Supplementary material for: Job performance among health professionals in Ethiopia: a systematic review and meta-analysis
Source: Public Health Rev. 2026 Jun 17;47:1609470. doi: 10.3389/phrs.2026.1609470 (PMC13318800; doi:10.3389/phrs.2026.1609470)
Supplement: Supplementary file 3 [file Supplementaryfile2.docx]

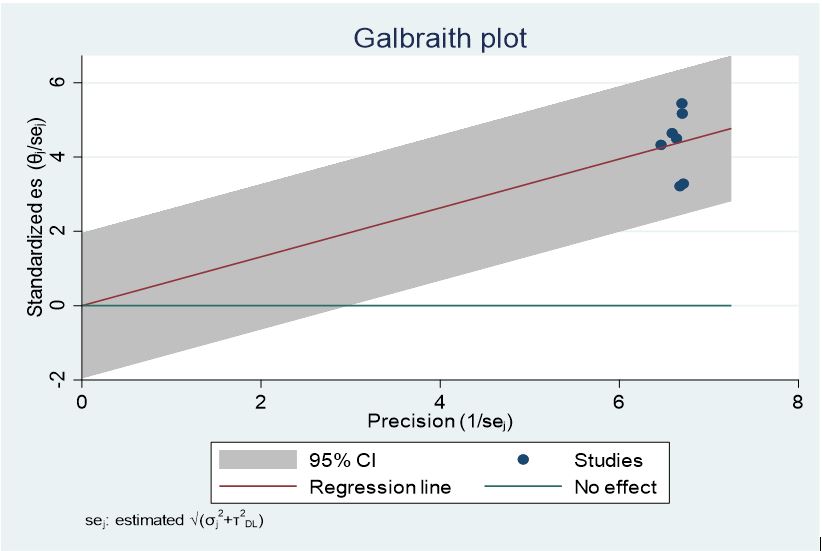


Figure 1: Galbraith plot of the pooled estimate of good job performance among health professionals in Ethiopia
