# Supplementary material for: Job performance among health professionals in Ethiopia: a systematic review and meta-analysis
Source: Public Health Rev. 2026 Jun 17;47:1609470. doi: 10.3389/phrs.2026.1609470 (PMC13318800; doi:10.3389/phrs.2026.1609470)
Supplement: Supplementary file 4 [file Supplementaryfile4.docx]

Factors associated with good job performance among health professionals in Ethiopia

| Associated factors | AOR with 95% CI | Direction of association | Author, publication year |
| --- | --- | --- | --- |
| Agree that there is sufficient staff | 1.7(1.19, 2.44 | Positive | Daba L etal, 2024 (38) |
| Agree that remuneration is according to experience | 1.89(1.35, 2.67) | Positive | Daba L etal, 2024 (38) |
| Agree that I find my work rewarding | 1.5(1.01, 2.23) | Positive | Daba L etal, 2024 (38) |
| Agree that objectives to be achieved are known by individuals to be assessed | 1.88(1.32, 2.67) | Positive | Daba L etal, 2024 (38) |
| Agree that feedback on how the staff is performing is provided throughout the year | 1.65(1.17, 2.33) | Positive | Daba L etal, 2024 (38) |
| Perceive feedback on performance appraisal is good | 4.551(1.848, 11.210) | Positive | Tesfaye T etal, 2015 (37) |
| Having good self-rated knowledge and skill | 14.02(6.351, 30.947) | Positive | Tesfaye T etal, 2015 (37) |
| Female sex | 1.896(1.13, 3.18) | Positive | Ousman Y etal, 2023 (10) |
| Marital status (married) | 1.911(1.116, 3.270) | Positive | Ousman Y etal, 2023 (10) |
| Working hour more than 8 hours | 2.91(1.653, 5.123) | Positive | Ousman Y etal, 2023 (10) |
| Good working condition | 2.164(1.218, 3.844) | Positive | Ousman Y etal, 2023 (10) |
| Satisfaction with job | 2.48(1.274, 4.476) | Positive | Ousman Y etal, 2023 (10) |
| Profession type-General Practitioner | 4.832(1.213, 19.240) | Positive | Bewket AG etal, 2023 (35) |
| Having good motivation | 2.458(1.322, 4.568) | Positive | Bewket AG etal, 2023 (35) |
